# Optic nerve sheath diameter by bedside ultrasound is a reliable screening test for cerebral edema in the comatose ICU patient

**DOI:** 10.1186/cc14537

**Published:** 2015-03-16

**Authors:** A Mohamed, N Alharbi, N Salahuddin, I Hussain, O Solaiman

**Affiliations:** 1 King Faisal Specialist Hospital & Research Centre, Riyadh, Saudi Arabia

## Introduction

ICU patients may remain comatose after resolution of critical illness. Frequently this is due to delayed sedative clearance but may also result from increases in intracranial pressure (ICP) and cerebral edema. We proposed that measurement of the optic nerve sheath diameter (ONSD) is a rapid, bedside screening test that can be used to quickly identify patients with cerebral edema and increased ICP.

## Methods

This was a prospective, observational study carried on consecutive patients admitted to a multidisciplinary medical and surgical ICU. Stable patients with unexplained coma and scheduled for brain imaging were included. Patients with obvious ocular trauma or on sedative, narcotic infusions were excluded. ONSD was measured using a 7.5 to 10 MHz linear array ultrasound transducer probe placed on the closed eye in the transverse axis. The ONSD was measured at a predefined point 3 mm posterior to the globe. Both eyes were measured and the mean value used. The study protocol was approved by the Hospital Research Ethics Committee (RAC Prop No. 2141 103). Statistical analysis was carried out using SPSS version 20.0.

## Results

ONSD was measured in 43 patients; mean age was 62 ± 19.2 years, 48% (*n = *20) were female. Admitting diagnosis was sepsis in 24% (*n = *10), intracranial vascular event in 21% (*n = *9), cardiac arrest in 12% (*n = *5), hepatic encephalopathy in 7% (*n = *3), malignancy with metastases in 7% (*n = *3) and other causes in 28% (*n = *12). The ONSD measured correlated highly between eyes, Spearman's ρ = 0.799, *P ≤*0.001. The area under the ROC curve for detecting cerebral edema was 0.812 (95% CI = 0.667 to 0.957). Using a 0.58 cm cutoff ONSD diameter, the sensitivity was 86%, specificity 74%, negative predictive value 96% and the positive likelihood ratio = 3.3.

## Conclusion

This study suggests that bedside measurement of ONSD by ultrasound performs well as an initial screening test for cerebral edema. The identified cutoff value of 0.58 cm can be used to detect cerebral edema with reasonable accuracy.

